# Aging: Environmental Threats to Elders’ Neurologic Health

**Published:** 2009-01

**Authors:** Julia R. Barrett

Age-related chronic diseases will put unprecedented stress on U.S. society with a near-doubling of the number of people aged 65 years and older by 2030, according to the U.S. Administration on Aging. These diseases are also complex. An October 2008 report, *Environmental Threats to Healthy Aging: With a Closer Look at Alzheimer’s & Parkinson’s Diseases*, now describes in greater detail how a lifetime of environmental factors from conception onward shapes our health in our later years.

The report, published by the Greater Boston Physicians for Social Responsibility and the Science and Environmental Health Network, uses a broad definition of *environment*, encompassing the physical, biological, social, and cultural contexts in which our lives are rooted. These contexts influence biological pathways that determine various outcomes, from good health in old age to chronic health issues including cardiovascular disease, diabetes, obesity, metabolic syndrome, and neurodegenerative disorders such as Alzheimer disease (AD) and Parkinson disease (PD). The authors suggest that environmental improvements, from our diet to our access to quality health care, could reduce, delay, or someday perhaps prevent diseases associated with aging.

“I think this report is exceptionally well done and valuable,” says Peter J. Whitehouse, a professor of neurology at Case Western Reserve University. “It will contribute in a major way to the reframing of AD, which desperately needs it.”

But the inclusion of neurodegenerative diseases among potentially preventable diseases gives pause to some researchers. “It’s tempting to believe what they’re saying with regard to AD and PD, but it has not been proven to be true,” says Robert Butler, president and CEO of the nonprofit International Longevity Center–USA in New York City. “I applaud the concept of what they’ve done but with the proviso that it has not yet been established.”

The report authors are confident that the possibility for prevention exists, though. They began their analysis with an interest in neurodegenerative diseases, but they quickly realized that the underlying mechanisms mirrored those involved in a suite of other disorders, including diabetes, obesity, metabolic syndrome, and cardiovascular disease—the so-called Western disease cluster. Those disorders share common biological pathways such as up-regulation of inflammation and oxidative stress and disruption of insulin signaling.

Citing numerous published studies, the authors describe multiple ways in which the environment feeds into these pathways and ultimately into the Western disease cluster. Likewise, environmental factors that act via these pathways could promote neurodegenerative diseases, says coauthor Ted Schettler, science director of the Science and Environmental Health Network, a consortium of U.S. environmental groups.

For example, he says there is ample evidence that oxidative stress and inflammation contribute to dopaminergic neuron loss in the substantia nigra, a hallmark of PD. “And given the strong body of evidence finding pesticides to be a risk factor for PD, along with mechanistic data from animal [studies] showing the role of oxidative stress and inflammation, our conclusions are not mere speculation but are based on several lines of evidence,” he says. Additionally, conditions such as diabetes and obesity are associated with increased risk for dementia and cognitive decline.

“We should begin to think of cognitive decline and AD as being related to the same causal pathways that are leading into obesity, diabetes, and cardiovascular disease, which are so prevalent in today’s society,” says Schettler. “Our conclusion is that we’re sitting at the cusp of an explosion of neurodegenerative and related diseases, not only because of an aging population but also because we have set the stage through all of the things that are contributing to diabetes, obesity, and cardiovascular disease.”

To meet and perhaps prevent the anticipated surge in age-related disease incidence, the authors outline potential cross-cutting solutions. Communities need to bolster access to healthful foods, which encompasses everything from providing business opportunities for groceries and food co-ops to supporting nursing mothers. Communities also need to support planning and development that incorporate green spaces and parks, public transport, and pedestrian and bicyclist safety.

Nationally, farm policies need to support sustainable production of healthful foods. Meaningful federal oversight is needed for phasing out known toxics, acting on early warnings of adverse effects, and keeping the public informed and protected. However, the authors emphasize, strategies cannot be adopted in isolation from one another with the expectation that they’ll be successful.

Julie Andersen, a professor at the nonprofit Buck Institute for Age Research in Novato, California, notes that the report consolidates information in a way that’s accessible to an educated lay audience. “The authors present information that is probably unknown to researchers outside specific areas and that almost certainly has not reached health care providers,” she says.

S. Jay Olshansky, a professor of epidemiology at the University of Illinois at Chicago School of Public Health, adds that the authors bring to the forefront the concept that we are capable of creating a better environment for healthy aging. “There’s a lot of low-hanging fruit,” he says. “We just need to pick it.”

## Figures and Tables

**Figure:**
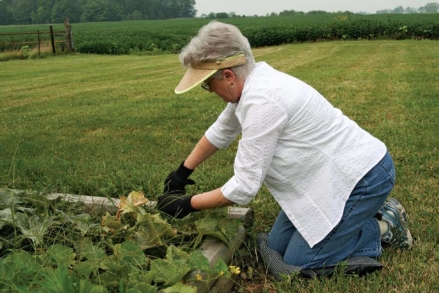
Health in later years depends on a lifetime’s worth of environmental factors.

